# Psychological responses to interval and continuous exercise in people living with HIV: A single‐blind, counterbalanced, crossover study

**DOI:** 10.1111/hiv.70193

**Published:** 2026-01-19

**Authors:** Phelipe Wilde, Victor S. de Queiros, Jason R. Jaggers, Angelo Sabag, Júlio M. Alves, Elaine Fernandes, Roberto F. C. Rocha, Paulo F. de Almeida‐Neto, Ronaldo Vagner Thomatieli‐Santos, Paulo Moreira Silva Dantas

**Affiliations:** ^1^ Centro de Ciências da Saúde Universidade Federal do Rio Grande do Norte Natal Brazil; ^2^ Departamento de Educação Física Universidade Estadual da Paraíba (UEPB) Campina Grande Brazil; ^3^ Department of Health & Sport Sciences University of Louisville Kentucky Louisville USA; ^4^ Sydney School of Health Sciences The University of Sydney Sydney New South Wales Australia; ^5^ Departamento de Biociências Universidade Federal de São Paulo São Paulo Brazil; ^6^ Departamento de Educação Física Universidade Federal do Rio Grande do Norte Natal Brazil

**Keywords:** acquired immunodeficiency syndrome, affective responses, exercise preference, HIIT

## Abstract

**Aim:**

This study compared acute psychological responses to a single session of low‐volume high‐intensity interval exercise (HIIE‐LV), high‐volume high‐intensity interval exercise (HIIE‐HV), and moderate‐intensity continuous exercise (MICE) in people living with HIV, and healthy controls using a randomized, counterbalanced crossover design.

**Methods:**

The participants (people living with HIV, and healthy controls) completed three exercise sessions in randomized order: HIIE‐HV (4 × 4 min at 80% of maximal power output [W_max_]), HIIE‐LV (10 × 60 s at 90% W_max_), and MICE (30 min at 60% W_max_). Psychological outcomes included affective response assessed by the Feeling Scale, exercise enjoyment and future exercise intention (FEI), while rating of perceived exertion (RPE) was recorded throughout the exercise. Data were analysed using repeated‐measures ANOVA with the group as a between‐subject factor.

**Results:**

All participants completed the three exercise conditions and were included in the analyses (11 people living with HIV and 11 healthy controls). In people living with HIV, exercise enjoyment was higher following HIIE‐HV compared with healthy controls (*p* = 0.031). No between‐condition differences were observed for affective response or FEI in people living with HIV. During exercise, affective responses did not differ between exercise modalities in people living with HIV, whereas healthy controls reported lower affective responses during HIIE‐HV compared with HIIE‐LV and MICE. RPE was significantly higher during HIIE‐HV compared with HIIE‐LV and MICE in both groups (*p* < 0.05).

**Conclusion:**

People living with HIV demonstrated similar affective responses and FEI following MICE and HIIE compared with healthy adults, despite higher perceived exertion during HIIE‐HV. Notably, people living with HIV reported higher exercise enjoyment following HIIE‐HV, suggesting that this exercise modality may be particularly well tolerated and positively perceived in this population.

## INTRODUCTION

With the advancement of antiretroviral therapies (ART), people living with HIV have achieved better control of viral load, resulting in a healthier immune system, reduced mortality rates, and increased life expectancy [1, 2, 3, 4]. However, people living with HIV still exhibit higher premature mortality rates compared to the general population, and are ageing with a higher prevalence of non‐communicable chronic diseases [5, 6, 7, 8, 9, 10]. As a result, adjunct therapies such as physical exercise have been recommended to assist in pharmacological treatment and improve the health status of this population [11, 12].

Although the benefits of physical exercise for people living with HIV are widely recognized, there is still a high dropout rate in physical training programmes among this population. Several factors, such as the lack of professional supervision, motivation, aerobic training programme, as well as moderate frequency and intensity, have been shown to influence the abandonment of these training programmes [13]. Dropout hampers long‐term health outcomes significantly, as sustained exercise adherence is crucial for managing HIV‐related complications and improving overall quality of life. Additionally, according to a meta‐analysis by Vancampfort et al., only 50.7% of people living with HIV meet the recommendation of 150 min of moderate‐intensity physical activity per week. Furthermore, studies have shown that people living with HIV spend only 98.9 min per day being physically active, which is less than other populations with chronic diseases [14]. Additionally, dropout rates of physical activity interventions are much higher in people living with HIV than in many other populations with chronic morbidities, necessitating the development of new exercise formats tailored to the needs of people living with HIV to improve adherence and health outcomes [13].

Due to low adherence rates and physical inactivity behaviours, it is essential to identify the training parameters that elicit negative affective responses, as these may impact motivation and adherence. Addressing these factors could ultimately enhance the engagement of people living with HIV in physical exercise programmes. High‐Intensity Interval Training/Exercise (HIIE) has been investigated as an alternative to promote a greater “feel‐better” sensation, and in the last 12 years, more than 200 articles have been published investigating exercise enjoyment and/or affective responses during HIIE [15]. However, none of these studies included people living with HIV among the populations being tested.

Given the lack of studies and concrete evidence on which exercise strategies can promote adherence among people living with HIV, investigating affective responses, exercise enjoyment, and future exercise intentions following different volumes of HIIE may help identify how this population psychologically responds to HIIE. This is essential for developing strategies aimed at sustaining participation in physical training programmes within this population. Therefore, the objective of the present study is to investigate the acute effects of HIIE on psychological parameters in people living with HIV.

## MATERIALS AND METHODS

The present study was conducted as a single‐blind, counterbalanced, crossover design involving men living with HIV and men without HIV (healthy controls). Participants were recruited between September 2023 and February 2024. Participants were randomly allocated into one of three groups using a randomization tool and then assigned in a counterbalanced manner to perform three different exercise sessions: Low‐Volume High‐Intensity Interval Exercise (HIIE‐LV), High‐Volume High‐Intensity Interval Exercise (HIIE‐HV), and Moderate‐Intensity Continuous Exercise (MICE). Each participant completed the three sessions in one of six possible sequences to ensure counterbalancing: (1) HIIE‐HV > MICE > HIIE‐LV, (2) HIIE‐HV > HIIE‐LV > MICE, (3) HIIE‐LV > HIIE‐HV > MICE, (4) HIIE‐LV > MICE > HIIE‐HV, (5) MICE > HIIE‐LV > HIIE‐HV, and (6) MICE > HIIE‐HV > HIIE‐LV. The counterbalanced randomization was performed using the JAMOVI® software (version 2.3.18, Sydney, Australia) with the “Randomizer” module available in its software library. Randomization was performed by one of the researchers who did not participate in the application of the exercise or data analysis. The individuals were recruited from a social project at the Universidade Federal do Rio Grande do Norte, which provides physical activity programmes for people living with HIV, as well as from the Hospital Giselda Trigueiro and the Serviço de Atendimento Especializado in Natal. Health controls were recruited through research advertisements on social media.

The study received approval from the Research Ethics Committee (5176786) of the UFRN and was registered on the Open Science Framework Registries platform (DOI: 10.17605/OSF.IO/V42QR). All research procedures, as well as their potential risks and benefits, were presented and explained in detail to the subjects. Following their agreement to participate, all participants signed an Informed Consent Form. The principles set out in the Declaration of Helsinki were followed. All participant data is confidential and non‐identifiable. This study was conducted in accordance with the CONSORT 2025 checklist guidelines for randomized controlled trials.

### Inclusion and exclusion criteria

Men living with HIV aged 20–45 years, who had been receiving antiretroviral therapy for at least 6 months were eligible to participate. All participants were required to provide medical clearance (for exercise) issued by a physician responsible for their clinical follow‐up at the Specialized Care Service or the referral hospital. Participants were considered physically active if they met the criteria for being classified as physically active according to the short version of the International Physical Activity Questionnaire (IPAQ), indicating engagement in moderate‐ and/or vigorous‐intensity physical activity during the 4 weeks preceding study enrolment. Individuals with osteomuscular limitations, a history of cardiovascular disease, illicit drug use, smoking, alcohol abuse, or non‐compliance with assessment recommendations were excluded. The same inclusion and exclusion criteria were applied to healthy controls, except for the HIV‐related clinical criteria.

### Assessments and study procedure

For sample characterization, evaluations of body composition and cardiorespiratory capacity were performed 1 week before the exercise session. Subject participation was carried out over 4 weeks, divided as follows: Week 1: initial screening with anamnesis application, physical activity level assessment, body composition evaluation, and maximal exercise test; Weeks 2, 3, and 4: performance of the HIIE‐LV, HIIE‐HV, or MICE exercise protocol and respective psychophysiological assessments. The application of the scales 10 min after the end of the exercise was carried out by a researcher who was not in the session, and who did not know which session had been applied at that time. The primary outcome of the study was affective response during exercise, assessed using the Feeling Scale. Secondary outcomes included exercise enjoyment, FEI and RPE.

### Sample characterization

Demographic characteristics including personal data, past and current medical history, time since HIV diagnosis, and duration of antiretroviral therapy use were collected. Physical activity level was assessed using the short version of the IPAQ [16]. Body fat was evaluated using the indirect method of Dual‐Energy X‐Ray Absorptiometry (DXA) (GE Healthcare® Lunar Prodigy Advance, Madison, USA).

### Maximal exercise test

All participants were informed in advance to abstain from alcohol consumption and intense physical activities for at least 48 h prior to the maximal exercise test. Participants were also instructed to avoid energy drinks on the day of testing, encouraged to adhere to good sleep hygiene practices the night before the assessment, and refrain from eating in the last 2 h before the assessment. Immediately prior to the maximal exercise test, participants were familiarized with the RPE scale, which was also administered during the exercise sessions. Heart rate (HR) was assessed using a HR monitor (Polar H10, Polar Electro Oy, Kempele, Finland) [17], and resting blood pressure was measured (model HEM‐7200‐Omron). After familiarization with the RPE scale, the maximal exercise test was performed on an electric cycle ergometer (model CG04, Inbrasport, Porto Alegre, Brazil), with 1 W precision and computer‐controlled magnetic load. Ventilatory variables were measured using a metabolic gas analyzer (Quark CPET, Cosmed, Italy) utilizing the breath‐by‐breath method, with averages calculated every 20 s. Before each test, the gas analysers and turbine flow meter system were calibrated following the manufacturer's instructions, using a calibrated 3.0‐L syringe with a known gas mixture concentration (FO_2_: 0.16; FCO_2_: 0.05; N_2_ as equilibrium).

The maximum incremental test began with an initial workload of 65 W and increased by 25 W every 2.5 min, with a cycling speed of 55–65 rpm/min [18]. The test was stopped when participants were unable to maintain cycling cadence (<50 rpm/min) for 5 s or more or due to voluntary exhaustion. The accepted criteria for maximum effort included volitional fatigue, a respiratory exchange ratio >1.1, and HR >95% of the maximum predicted based on age (220 − age). RPE was recorded at the end of each minute during the test, while HR and VO_2_ were recorded throughout the exercise. Maximum watts (W_max_) was recorded for use in the determination of intensity for exercise prescription.

### Psychological measures

#### Rating of perceived exertion

RPE was assessed using the Borg 6–20 scale, where 6 represented the lowest intensity or no effort, and 20 represented the highest perceived exercise intensity [19]. The scale was administered in accordance with recommendations described by Pageaux [20]. Perceived exertion was recorded during the final 10 seconds of each high‐intensity interval in the HIIE‐LV group, totalling 10 measures, and every 2 min in the HIIE‐HV group, totalling 8 measures. In the MICE group, perceived exertion was measured every 3 min, totalling 10 measures.

#### Affect

Affective response was assessed using the Feeling Scale, a validated single‐item, 11‐point bipolar scale ranging from −5 to +5, with the following verbal anchors: very bad (−5), bad (−3), somewhat bad (−1), neutral (0), reasonably good (+1), good (+3) and very good (+5) [21]. The scale was administered during the exercise sessions and again 10 min after exercise completion. Participants received standardized instructions during the initial screening, and in‐exercise assessments were conducted at the same time points as RPE.

#### Exercise enjoyment

To assess enjoyment, participants used a single‐item measure on a 7‐point rating scale ranging from 1 (not at all) to 7 (extremely) to respond to the item: ‘Use the following scale to indicate how much you enjoyed this exercise session.’ The scale was applied 10 min after the end of the experimental session [22]. The scale was introduced to participants during the week prior to the exercise and immediately before the session.

#### Future exercise intention (FEI)

Participants were asked 10 min after completing the exercise about their future intention to engage in exercises performed in the laboratory. A future intention probability scale was used for this purpose. The scale consisted of a single item ranging from 0% to 100% in 10% increments [23]. Participants were asked to report on the scale in response to the following question: ‘How interested are you in participating in an exercise program similar to the one you just completed in the next 2 weeks?’ The scale was introduced to participants during the week prior to the exercise and immediately before the session.

### Physical exercise protocol

To prescribe the three exercise protocols, we used the W_max_ related to the maximum VO_2_ (VO_2max_) obtained during the maximal exercise test to set the intensity levels. In the MICE protocol, participants engaged in continuous exercise at 60% of W_max_ for 30 min. The HIIE‐HV protocol included a 2.5 min warm‐up at 50% of W_max_, followed by four 4‐min high‐intensity intervals at 80% of W_max_, with 3 min passive recovery periods, and a 2.5 min cooldown at 50% of W_max_. The HIIE‐LV protocol involved a 1‐minute warm‐up at 50% of W_max_, ten 60 s high‐intensity intervals at 90% of Wmax with 60 s passive recovery periods, and a 1‐min cooldown at 50% of W_max_. Monitoring of heart rate occurred throughout all exercise sessions. To facilitate comparison across exercise modalities, an overview of the structure and prescribed intensity of each exercise protocol, including warm‐up and cool‐down phases, is provided in Table 1.

**TABLE 1:** Characteristics of exercise protocols.

Protocol	Total duration	Work interval	Intensity (%W_max_)	Recovery	Warm‐up and cool‐down	Session load
HIIE‐LV	21 min	10 × 60 s	90% W_max_	60 s passive	1 min at 50% W_max_	Low volume (<15 min)
HIIE‐HV	30 min	4 × 4 min	80% W_max_	3 min passive	2.5 min at 50% W_max_	High volume (≥15 min)
MICE	30 min	Continuous	60% W_max_	‐	‐	‐

Adverse events were monitored throughout the study based on participant self‐reports during and after each exercise session, as well as spontaneous reports of symptoms in the days following the sessions.

### Statistical analysis

Data normality was verified using the Shapiro–Wilk test (Table S1). Descriptive data for participant characteristics are presented as mean ± standard deviation and median (Q1–Q3). Baseline characteristics of people living with HIV and healthy controls were compared using independent‐samples *t*‐tests.

Normality analyses indicated a normal distribution for average HR, average power output in watts (W), energy expenditure (kcal), VO_2_, and psychological measures assessed during exercise, including affective response and RPE. However, psychological responses assessed after exercise, including FEI, exercise enjoyment, and affective response, did not follow a normal distribution. Bonferroni‐adjusted post hoc tests were conducted to identify specific differences between groups.

Two‐way ANOVA was employed to analyse the effects of the interventions on the means of HR, Wmax, kcal, VO_2_, FEI, exercise enjoyment, and affective response assessed after exercise [24]. Repeated‐measures ANOVA was applied to assess the effects of the interventions on the means of RPE and affective responses during exercise [24]. A significance level of *p* < 0.05 was adopted for all analyses, which were performed using Jamovi software (version 2.3.18, Sydney, Australia).

### Sample size

Sample size estimation was performed using GPower software (version 3.1.9.7), based on an effect size of 0.82 reported by Stork, Gibala and Ginis [25], for peak negative affect during single sessions of high‐intensity interval exercise and moderate‐intensity continuous exercise. Assuming an alpha level of 0.05 and a statistical power of 0.80, the minimum required sample size was 12 participants. To account for a potential dropout rate of 20%, a total sample size of 15 participants was planned. However, the study was completed with 11 participants due to logistical constraints that prevented continuation of data collection. A post hoc power analysis was subsequently conducted using GPower (version 3.1.9.7) for a repeated‐measures ANOVA with a within‐between interaction, employing the F statistic as the effect size measure. The analysis indicated a power of 0.335 for the interaction between exercise modality and condition, suggesting limited sensitivity to detect interaction effects with the final sample size. In contrast, the statistical power for the main effects of exercise modality and time was 1.000, indicating a high ability to detect these effects. This sample‐size shortfall should be viewed as a learning opportunity, highlighting the need for more robust sample sizes in future studies to enhance the detection of interaction effects. Consequently, while some outcomes remain exploratory, this insight provides valuable guidance for planning subsequent research with larger and more comprehensive cohorts.

## RESULTS

**FIGURE 1:**
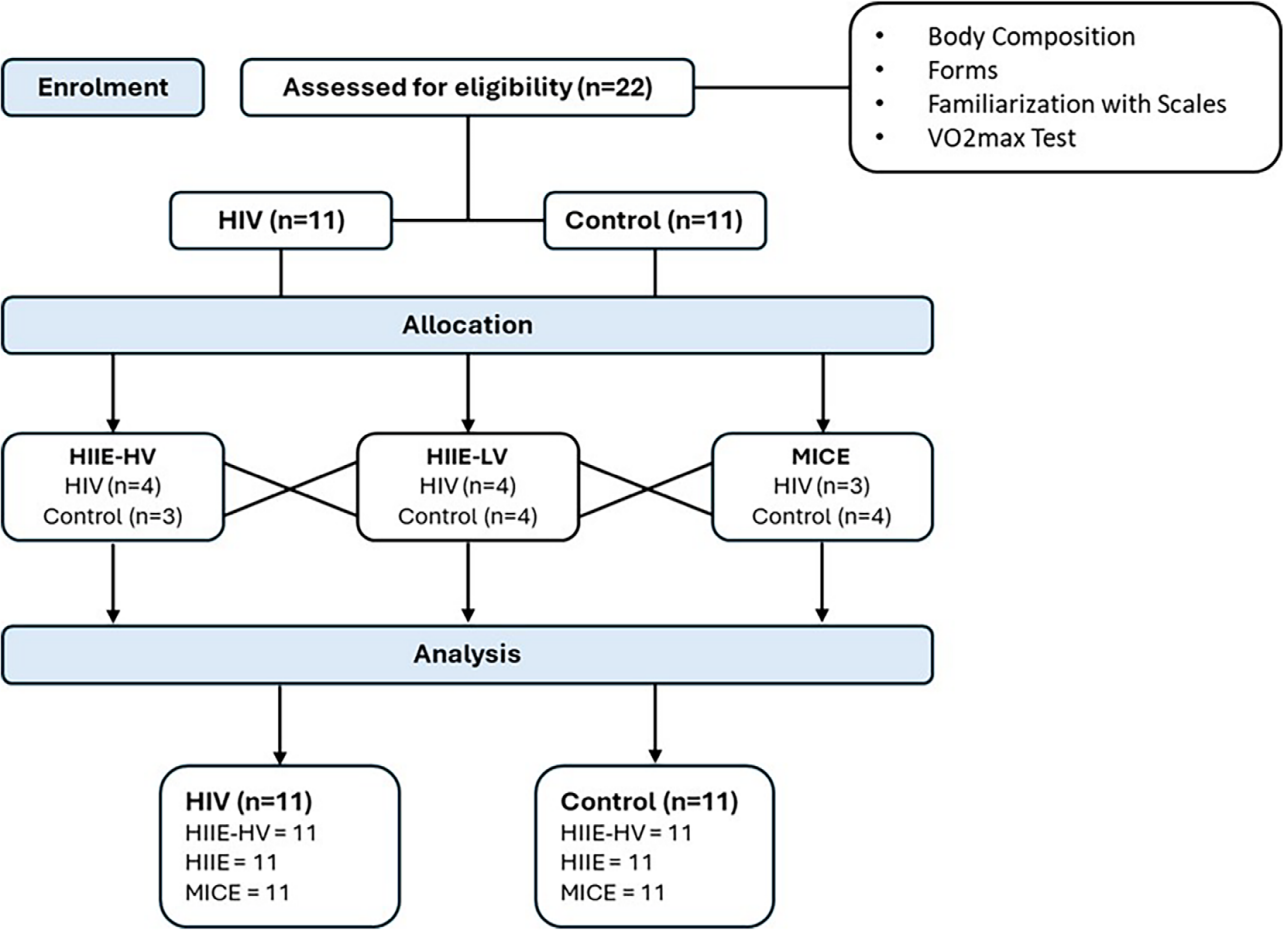
CONSORT 2025 flow diagram.

A total of 22 participants comprising 11 people living with HIV and 11 healthy controls were enrolled in the study. All participants completed the three‐exercise sessions and were included in the analyses. No adverse events were reported during or after any exercise session, and all participants completed the proposed exercise protocols without interruption. In addition, no participant reported symptoms of infection or excessive fatigue in the days following the exercise sessions. Participant flow through the study is presented in Figure 1 (CONSORT 2025 flow diagram), and participant characteristics are summarized in Table 2.

**TABLE 2:** Participant characteristics.

Variables	HIV (*n* = 11)		Control (*n* = 11)		*p*
	Mean ± SD	Media*n* (Q1‐Q3)	Mean ± SD	Media*n* (Q1‐Q3)	
Age (year)	34.4 ± 5.54	34 (30–37.5)	33.3 ± 5.92	34 (28.5–36)	0.660
Body mass (kg)	75.7 ± 9.67	75.7 (70.5–84.5)	79.3 ± 11.64	79.3 (70.5–85)	0.438
Fat mass (%)	26.5 ± 6.45	22.6 (22.1–31.9)	28.0 ± 4.90	29.1 (24.3–31.1)	0.546
BMI (kg·m^−2^)	25.8 ± 3.43	24.8 (23.8–28.4)	26.7 ± 3.09	26.4 (25–29.6)	0.542
HR_max_ (bpm)	174.3 ± 7.31	177 (169–181)	175 ± 10.22	173 (169–181)	0.850
W_max_ (W)	180.9 ± 35.83	190 (165–203)	203.6 ± 49.20	190 (178–215)	0.230
VO_2max_ (mL · kg^−1^ · min^−1^)	33.3 ± 6.20	30.8 (29.3–37.4)	35.9 ± 6.28	34.4 (31.4–38.9)	0.300
Physical activity (met)	6226.5 ± 6536.66	3276 (2487–8317)	6910.5 ± 8978.88	3292 (1877–6521)	0.870
HIV variables					
Diagnostic time (year)	7.2 ± 3.8				
ART time (year)	6.2 ± 3.1				
Viral load	Undetectable (100%)				
Type of ART					
NRTI + II	54.54% (6)				
NRTI	27.27% (3)				
NRTI + PI	9.09% (1)				
NRTI + INNTR	9.09% (1)				

*Note*: Values are presented as mean ± standard deviation (SD) and median (Q1‐Q3), and as frequency for antiretroviral medications.

Abbreviations: ART, antiretroviral therapy; BMI, body mass index; HRmax, maximum heart rate; II, integrase inhibitor; NRTI, nucleoside reverse transcriptase inhibitor; NNRTI, non‐nucleoside reverse transcriptase inhibitors; PI, protease inhibitors; VO_2_max, maximal oxygen uptake; Wmax, maximum power output.

### Physiological responses to exercise

The physiological responses associated with higher VO_2_, average watts, average HR, and Kcal are summarized in Table 3. Significant effects were observed across trials for VO_2_ (*F* = 18.96; *p* < 0.001; $\eta_{p}^{2}=0.387$), average watts (*F* = 26.86; *p* < 0.001; $\eta_{p}^{2}=0.472$), average HR (*F* = 15.8; *p* < 0.001; $\eta_{p}^{2}=0.346$), and Kcal (*F* = 33.99; *p* < 0.001; $\eta_{p}^{2}=0.535$). A higher response that was statistically significant was observed in the HIIE‐HV and HIIE‐LV groups compared to MICE for VO_2_, average watts and HR. In addition, we observed an increase in Kcal in the HIIE‐HV and MICE groups compared to HIIE‐LV.

**TABLE 3:** Physiological responses to exercise.

Variable	Condition	HIIE‐HV (*n* = 11)	HIIE‐LV (*n*‐11)	MICE (*n* = 11)
V0_2_ (mL/min/kg)	HIV	28.2 ± 3.61* ^,^ ^†^	25.0 ± 3.33^§^ ^,^ ^ǂ^	21.2 ± 3.54
	Control	27.6 ± 3.52* ^,^ ^†^	24.9 ± 4.66^§^ ^,^ ^ǂ^	21.0 ± 3.36
Average Watts (W)	HIV	143.1 ± 31.5*	164.7 ± 35.8^§^ ^,^ ^ǂ^	99.9 ± 23.7
	Control	158.7 ± 32.7* ^,^ ^†^	182.2 ± 37.3^§^ ^,^ ^ǂ^ ^,^ ^˄^	111.3 ± 24.0
Average HR (bpm)	HIV	146 ± 9.04* ^,^ ^†^	142 ± 9.64^§^ ^,^ ^ǂ^	129 ± 10.76
	Control	149 ± 15.32* ^,^ ^†^	139 ± 14.46^§^ ^,^ ^ǂ^	126 ± 10.47
Kcal	HIV	229 ± 33.9^!^ ^,^ ^˄^	157 ± 20.4	229 ± 39.6^§,^ ^ǂ^
	Control	232 ± 34.6^!^ ^,^ ^˄^	166 ± 27.0	241 ± 38.5^§,^ ^ǂ^

* Significant difference between HIIE‐HV and MICE.

^†^ Significant difference between HIIE‐HV and MICE of different conditions.

^§^ Significant difference between HIIE‐LV and MICE.

^ǂ^ Significant difference between HIIE‐LV and MICE of different conditions.

^!^ Significant difference between HIIE‐HV and HIIE‐LV.

^˄^ Significant difference between HIIE‐HV and HIIE‐LV of different conditions.

### Psychological responses after exercise

To facilitate readability, the term “condition” refers to the HIV and Control (healthy adults) conditions, while the term “method” refers to the exercise methods (HIIE‐HV, HIIE‐LV and MICE). The psychological responses related to FEI, exercise enjoyment, and affective responses are summarized in Table 4. A significant difference between conditions (people living with HIV vs. control group) was observed for exercise enjoyment following the HIIE‐HV session, with higher enjoyment reported in the HIV condition (*p* = 0.031), indicating that those with HIV are more likely to respond positively to HIIE‐HV. No differences were observed between HIV and control for FEI and affective responses.

**TABLE 4:** Psychological responses after exercise.

Variable	Condition	HIIE‐HV (*n* = 11)	HIIE‐LV (*n*‐11)	MICE (*n* = 11)	*F*	*p*	$n_{p}^{2}$
FEI	HIV	7.45 ± 3.11	7.36 ± 2.87	7.36 ± 2.66	0.929^a^ 0.443^b^ 0.555^c^	0.339^a^ 0.644^b^ 0.577^c^	0.015^a^ 0.014^b^ 0.018^c^
	Control	5.73 ± 3.20	7.45 ± 2.91	6.91 ± 2.84			
Exercise Enjoyment	HIV	5.18 ± 1.47^⸸^	4.82 ± 1.60	5.14 ± 1.34	4.690^a^ 0.200^b^ 1.920^c^	0.034^a^ 0.819^b^ 0.406^c^	0.070^a^ 0.006^b^ 0.027^c^
	Control	3.82 ± 1.47	4.64 ± 1.50	4.36 ± 1.29			
Affect	HIV	2.18 ± 2.32	2.91 ± 2.07	2.73 ± 1.79	0.128^a^ 1.309^b^ 0.225^c^	0.721^a^ 0.278^b^ 0.799^c^	0.002^a^ 0.042^b^ 0.007^c^
	Control	1.91 ± 2.17	3.18 ± 1.60	2.18 ± 2.32			

*Note*: Superscript letters (a, b, c) indicate the corresponding statistical effects in the *F*, *p*, and $\eta_{p}^{2}$ columns.

Abbreviation: FEI, future exercise intention.

^a^ Main effect of condition.

^b^ Main effect of method.

^c^ Main effect of interaction (condition × method). *F* = F statistic; *p* = *p* value; $\eta_{p}^{2}$, partial eta squared (effect size).

^⸸^ Significant difference between HIIE‐HV of different conditions.

### Psychological responses during physical exercise

To summarize the results, only statistically significant differences are emphasized. These include differences between exercise methods (HIIE‐HV, HIIE‐LV and MICE) at the same stage in the HIV and control group and differences between conditions (HIV and control group) in the same exercise method and stage.

#### Affect

For Affect, participants reported feeling progressively better as the exercise sessions continued. The data indicated an interaction between time and condition, suggesting that as both groups progressed through the session, their affective response increased over time. This was supported by the statistical analysis showing significant improvements in mood across the sessions (*F* = 27.31; *p* < 0.001; $\eta_{p}^{2}:0.313$) and noted an interaction between time and condition (*F* = 2.58; *p* = 0.003; $\eta_{p}^{2}:0.041$).

**FIGURE 2:**
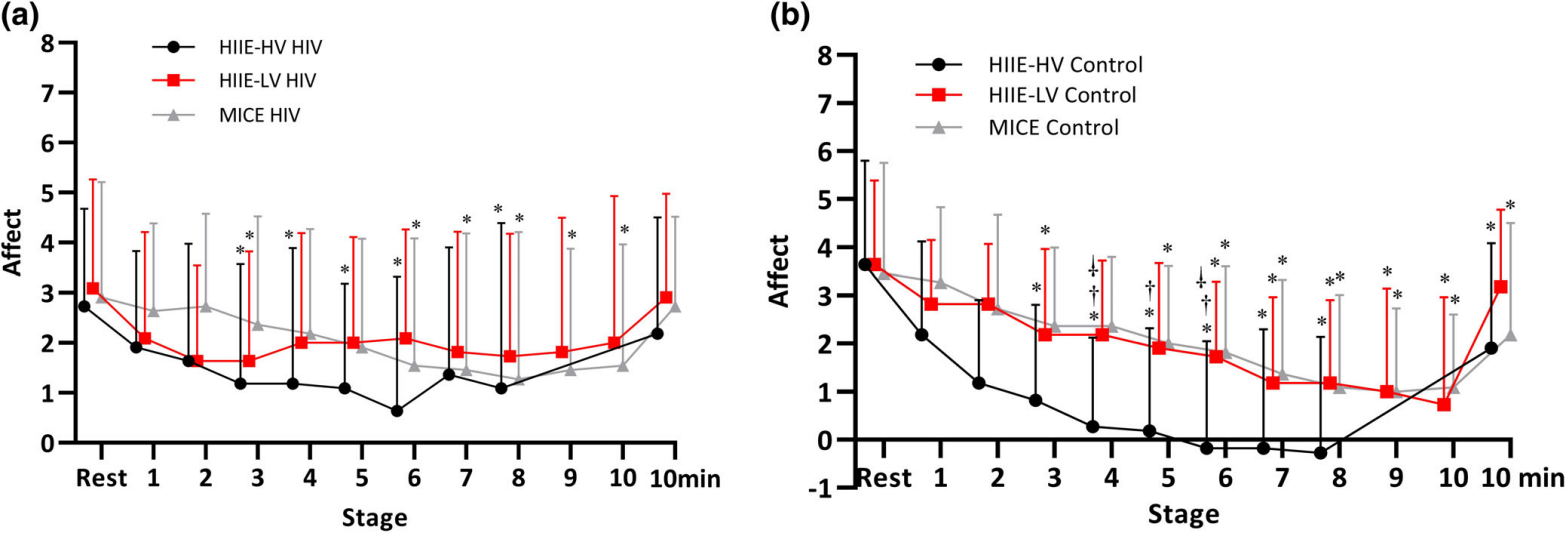
Affective responses during exercise. (a) Affective responses during exercise in people living with HIV. (b) Affective responses during exercise in healthy controls. Data are presented as mean and standard deviation. *Significantly different from rest; ^†^Significantly different between HIIE‐HV and MICE; ^⸸^Significantly different between HIIE‐HV and HIIE‐LV.

No statistically significant differences were observed between exercise methods at the same stage in the HIV condition, or between conditions (people living with HIV vs. control group) for the same method and stage. However, in the HIIE‐HV method applied to the control group, a significantly lower affective response score was observed at the first (Affect report 2), second (Affect report 4), and third stages (Affect reports 5 and 6) when compared to the HIIE‐LV method (Affect‐2 *p* = 0.044; Affect‐4 *p* = 0.030; Affect‐6 *p* = 0.046) and MICE (Affect‐4 *p* = 0.018; Affect‐5 *p* = 0.043; Affect‐6 *p* = 0.037) (see Figure 2). These findings suggest that healthy individuals may perceive differences in exercise intensities more distinctly when compared to people living with HIV.

#### Rating of perceived exertion

For RPE, an effect of time (*F* = 139.605; *p* < 0.001; $\eta_{p}^{2}:0.699$), exercise method (*F* = 3.36442; *p* = 0.041; $\eta_{p}^{2}:0.101$), and time × exercise method interaction (*F* = 204.494; *p* < 0.001; $\eta_{p}^{2}$) was observed.

**FIGURE 3:**
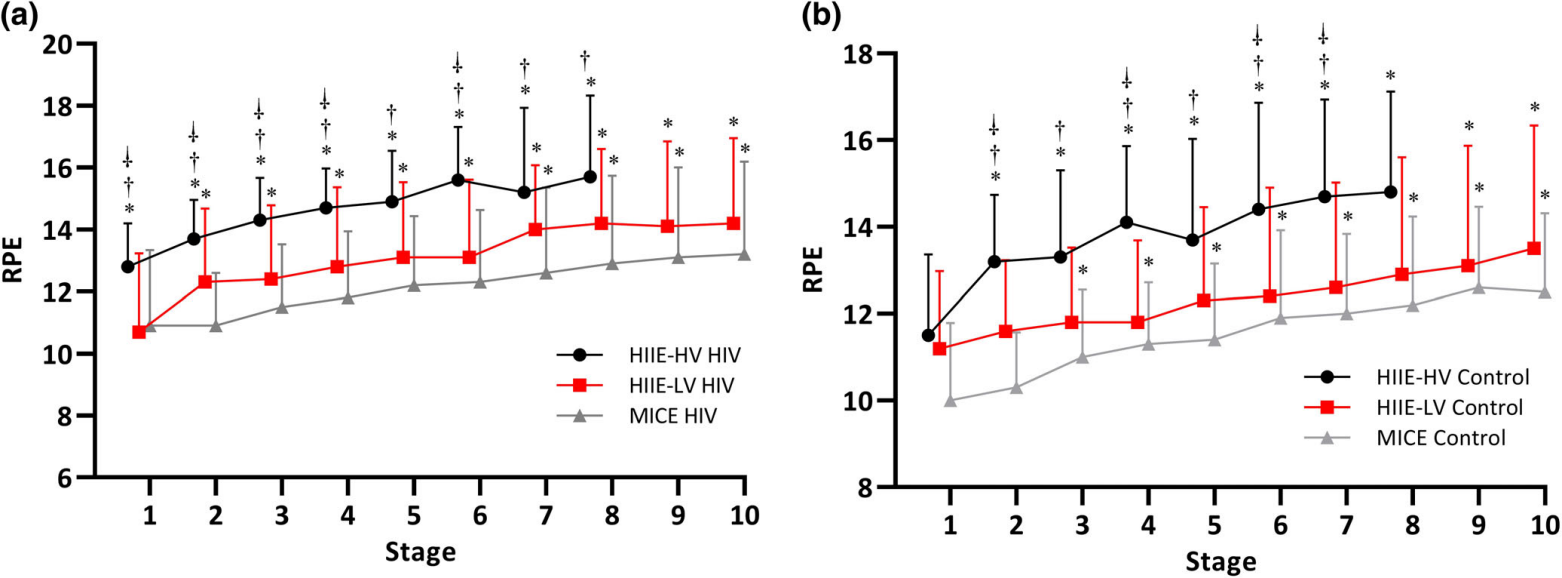
RPE responses during exercise. (a) RPE responses during exercise in people living with HIV. (b) RPE responses during exercise in healthy controls. Data are presented as mean and standard deviation. *Significantly different from rest; ^†^Significantly different between HIIE‐HV and MICE; ^⸸^Significantly different between HIIE‐HV and HIIE‐LV.

During the HIIE‐HV method in the HIV group, a significantly higher response was observed in the first (RPE reports 1 and 2), second (RPE reports 3 and 4), third (RPE reports 5 and 6) and fourth (RPE reports 7 and 8) stages compared with HIIE‐LV (RPE‐1 *p* = 0.017; RPE‐2 *p* = 0.046; RPE‐3 *p* = 0.020; RPE‐4 *p* = 0.022; RPE‐6 *p* = 0.011) and MICE (RPE‐1 *p* = 0.029; RPE‐2 *p* < 0.001; RPE‐3 *p* = 0.001; RPE‐4 *p* < 0.001; RPE‐5 *p* = 0.004; RPE‐6 *p* = 0.001; RPE‐7 *p* = 0.012; RPE‐8 *p* = 0.011) (see Figure 3). Similar patterns were observed in the control group, with significant differences across stages compared with HIIE‐LV and MICE.

## DISCUSSION

The main objective of this study was to analyse the acute effects of HIIE‐HV, HIIE‐LV and MICE on psychological responses known to enhance exercise adherence (affective response, enjoyment, perceived exertion and future exercise intention) in people living with HIV and healthy individuals. Affective response and future exercise intention assessed 10 min post‐exercise did not differ between the exercise methods tested for people living with HIV or healthy individuals. This finding was also observed for affective response monitored during exercise in the people living with HIV group but not among the healthy individuals. In contrast, exercise enjoyment, also assessed 10 minutes after exercise, showed higher values in the people living with HIV group in the HIIE‐HV. Moreover, in the healthy group, a lower effective response was recorded during specific moments of the HIIE‐HV session. In both groups, perceived exertion was higher during HIIE‐HV when compared with the HIIE‐LV and MICE. These findings present important considerations for exercise professionals when prescribing structured exercise to people living with HIV.

A robust body of evidence indicates that high‐intensity exercise can elicit more negative affective responses. The inverse relationship between exercise intensity and affective responses was demonstrated in the study by Ekkekakis et al. [26] which observed that exercises performed at intensities above the ventilatory threshold (VT) resulted in lower affective responses compared to those performed below the VT. Partially, our findings diverge from the results reported by Ekkekakis et al. [26] as no statistically significant differences were found in the current study between HIIE‐LV and MICE, either in the HIV group or the healthy group [26]. Unlike Ekkekakis et al. [26] who employed a continuous 15‐minute protocol to analyse the effect of intensity, the current study used an intermittent protocol (HIIE‐LV). Although HIIE‐LV involves higher intensity, its intermittent nature may have contributed to less negative affective responses, comparable to those observed with MICE [27].

Several studies have compared affective responses between HIIE and MICE; however, making direct comparisons across studies is highly challenging due to the substantial heterogeneity in the HIIE and MICE protocols tested. Using a HIIE‐LV protocol similar to the present study (10 intervals of 1 min at 100% W_peak_, interspersed with 1 min of active recovery at 20% W_peak_), Jung et al. (2014) observed a more positive affective response during MICE compared to HIIE‐LV at 2.5%, 42.5%,and 92.5% of the exercise session, which contrasts with our findings [28]. This discrepancy may be explained by differences in exercise intensity, as Jung et al. applied a higher intensity for HIIE‐LV and a lower intensity for MICE (~40% Wpeak) compared to the current study [28].

Similarly, Olney et al. [27] reported lower affective responses during HIIE‐LV (8 intervals of 1 min at 85% W_peak_, interspersed with 1 min of active recovery at 20% W_peak_) compared to MICE (40% W_peak_) at 50%, 75% and 100% of the exercise session. Although the intensity of HIIE‐LV was similar (85% vs. 90% W_peak_), the intensity used for MICE was lower than that in the present study. These findings support the notion that the higher intensity adopted for MICE (60% W_peak_) in the current study may account for the similar affective responses observed between this training model and HIIE‐LV.

As presented, two HIIE protocols were tested in our study. While HIIE‐LV elicited a similar affective response to MICE regardless of the group, HIIE‐HV resulted in a lower affective response compared to both HIIE‐LV and MICE in the healthy group, aligning with the findings of the aforementioned studies. This outcome is likely attributable to the greater physiological stress induced by HIIE‐HV, particularly due to the duration of the high‐intensity intervals, with interoceptive factors negatively influencing the affective response during this type of exercise. Notably, participants perceived HIIE‐HV as more intense, as reported in the RPE analyses (a variable inversely related to the affective response observed during HIIE sessions) [29].

Interestingly, in the HIV group, no significant differences in affective response were reported across the different exercise models, despite a significantly higher RPE during HIIE‐HV. It is important to note that, although no statistically significant difference was observed, a trend toward lower affective responses during HIIE‐HV can be identified at certain points of the exercise session. Research indicates that people living with HIV can experience approximately 20%–60% of cognitive symptoms with a high burden of HIV‐associated neurocognitive disorder (HAND) in the ART era, particularly in Sub‐Saharan Africa and Latin America [30, 31].We speculate that the lack of significant differences may be attributed to variability in the affective responses reported in the HIV group, which could be influenced by cognitive factors [26]. Although cognitive factors may influence affective responses to exercise, the potential role of HAND should be interpreted with caution. HAND was not assessed in the present study and therefore, any influence on affective responses remains speculative. Future studies including direct assessments of cognitive function are needed to clarify whether neurocognitive factors contribute to affective responses to HIIE in people living with HIV. Additionally, the sample size may have influenced the lack of significance.

To our knowledge, no previous studies have been published evaluating psychological responses to high‐ and moderate‐intensity aerobic exercise in people living with HIV. However, in a study by Nosrat et al. [32], resistance exercise performed at 100% of 10RM (3 sets of 10 repetitions with 90 s of rest between sets) across five strength training exercises (squat, chest press, lateral pulldown, dumbbell shoulder press and dumbbell biceps curl) resulted in lower affective responses, measured using the Feeling Scale, compared to moderate‐intensity resistance exercise at 70% of 10RM [32]. In the same study, RPE showed no statistically significant differences between the two intensity levels [32]. Although resistance training represents a different exercise modality, these findings differ from ours, where no differences in affective responses were found between exercise models in people living with HIV, and a significant increase in RPE was observed during HIIE‐HV in this population.

It is worth noting that all participants successfully completed the proposed exercise sessions, demonstrating the tolerability of these protocols in untrained healthy individuals and those with HIV. In contrast, a previous study comparing HIIE and continuous training in psychological responses reported that over 50% of the sample (8/15) were unable to complete the HIIE session due to high levels of fatigue [33]. The authors used intervals (~6.6) with a duration of 2 min at 100% VO_2_peak, interspersed with ~57 s (±10) of recovery. It is plausible that this work‐to‐recovery ratio (2:1) significantly increased muscular fatigue in the study by Oliveira et al. [33]. Further studies are needed to confirm whether the protocols adopted in our study have a greater tolerability capacity.

Evidence suggests that affective and behavioural responses to acute exercise sessions may influence subsequent exercise‐related behaviour, as psychological experiences assessed immediately after single exercise bouts have been associated with short‐term exercise choices and engagement in subsequent physical activity sessions [25]. In parallel, literature grounded in hedonic theory indicates that affective responses experienced during exercise are shaped by multiple individual and contextual factors, including perceived competence and self‐efficacy, environmental and social conditions, and the specific characteristics of the activity performed. Importantly, affective responses during exercise, particularly at moderate intensities, have been more consistently associated with future physical activity behaviour than post‐exercise affect, which tends to show weaker or inconsistent relationships with subsequent engagement [34]. Although prior physical activity history has not consistently moderated these associations, individual exercise experiences may still contribute to variability in affective and motivational responses. Accordingly, while the present study assessed responses to isolated exercise sessions, the higher post‐exercise pleasure observed following HIIE‐HV in people living with HIV may have practical relevance, as positive affective experiences during acute sessions could facilitate continued participation when similar exercise modalities are repeated over time. Nevertheless, longitudinal studies are required to determine whether these acute psychological responses translate into sustained exercise adherence in this population.

This study has limitations that must be considered when interpreting the findings. First, women were not included in the sample due to recruitment challenges at the study site; therefore, it remains unclear whether sex would have influenced the observed responses. Future research could intentionally oversample female people living with HIV to assess whether women's responses differ. Second, the study did not follow participants over an extended period, which limits the ability to infer whether acute psychological responses translate into long‐term exercise adherence, as a single exercise session and sustained participation are not equivalent. Future studies could adopt a longitudinal design to better understand the long‐term effects of different exercise modalities on adherence. Third, the sample size was relatively small. In addition, psychological outcomes were assessed using self‐reported and, in some cases, single‐item measures, which may limit sensitivity to subtle changes, and their psychometric properties have not been specifically established in people living with HIV. As strengths, this is the first study to evaluate psychological responses to high‐ and moderate‐intensity aerobic exercise in people living with HIV, providing an important foundation for future research on exercise adherence in this population. Moreover, the study employed a crossover, counterbalanced, and single‐blind design.

## CONCLUSION

People living with HIV exhibited responses similar to healthy male adults with respect to perceived exertion and affective response during exercise, as well as future exercise intention and affective response assessed 10 mins post‐exercise. Notably, affective responses during exercise did not differ between exercise modalities in people living with HIV, whereas healthy individuals reported lower affective responses during HIIE‐HV. In addition, people living with HIV reported greater post‐exercise enjoyment following HIIE‐HV compared with healthy controls. Overall, these findings indicate that people living with HIV respond comparably to MICE and HIIE when contrasted with healthy adults in an acute exercise context. However, these results should be interpreted with caution due to the limited sample size and the acute nature of the study design. Nevertheless, the present findings provide initial evidence that certain high‐intensity exercise modalities may be positively perceived by people living with HIV, and may have practical relevance for exercise prescription. Future studies should examine behavioural responses and exercise engagement over time to determine whether these acute psychological responses translate into sustained participation across different exercise modalities.

## CONFLICT OF INTEREST STATEMENT

None of the authors declare any conflicts of interest or financial involvement in the study.

## Supporting information


**Data S1.** Supporting Information.

## Data Availability

The data that support the findings of this study are available on request from the corresponding author. The data are not publicly available due to privacy or ethical restrictions.
