# Supplementary material for: Psychological responses to interval and continuous exercise in people living with HIV: A single‐blind, counterbalanced, crossover study
Source: HIV Med. 2026 Jan 19;27(4):616–27. doi: 10.1111/hiv.70193 (PMC13051039; doi:10.1111/hiv.70193)
Supplement: Supplementary file 1 — Data S1. Supporting Information. [file HIV-27-616-s001.docx]

**Table S-1. Shapiro-Wilk p-values.**

| **Variable** | **Group** | **Rest** | **1** | **2** | **3** | **4** | **5** | **6** | **7** | **8** | **9** | **10** | **10min** |
| --- | --- | --- | --- | --- | --- | --- | --- | --- | --- | --- | --- | --- | --- |
| Affect | HIIE-HV HIV | 0.016 | **0.126** | **0.509** | **0.202** | **0.129** | **0.202** | **0.602** | **0.775** | **0.426** | **-** | **-** | **0.126** |
|  | HIIE-LV HIV | 0.006 | 0.010 | 0.011 | **0.278** | **0.129** | **0.412** | **0.176** | **0.589** | **0.343** | **0.096** | **0.113** | 0.032 |
|  | MICE HIV | 0.003 | **0.185** | **0.126** | **0.236** | **0.348** | **0.176** | **0.250** | 0.041 | **0.426** | **0.136** | **0.392** | **0.269** |
|  | HIIE-HV Control | <0.001 | 0.011 | 0.026 | **0.070** | **0.738** | **0.204** | **0.788** | **0.410** | **0.115** | **-** | **-** | **0.667** |
|  | HIIE-LV Control | 0.006 | **0.379** | **0.498** | **0.699** | 0.015 | 0.015 | **0.482** | **0.699** | **0.846** | **0.246** | **0.547** | 0.046 |
|  | MICE Control | <0.001 | **0.061** | **0.188** | **0.207** | **0.128** | **0.169** | **0.295** | **0.165** | **0.351** | **0.296** | **0.278** | **0.479** |
| RPE | HIIE-HV HIV | **-** | **0.073** | 0.005 | **0.201** | **0.201** | 0.024 | **0.379** | **0.132** | **0.425** | **-** | **-** | **-** |
|  | HIIE-LV HIV | **-** | 0.011 | **0.174** | **0.318** | **0.276** | **0.645** | **0.767** | **0.176** | **0.946** | **0.866** | **0.853** | **-** |
|  | MICE HIV | **-** | **0.435** | **0.329** | **0.607** | **0.746** | **0.435** | **0.627** | **0.263** | **0.378** | **0.653** | **0.620** | **-** |
|  | HIIE-HV Control | **-** | **0.548** | **0.162** | **0.344** | **0.289** | **0.865** | **0.263** | **0.669** | **0.953** | **-** | **-** | **-** |
|  | HIIE-LV Control | **-** | **0.077** | 0.019 | 0.037 | 0.039 | **0.126** | **0.115** | **0.302** | **0.473** | **0.568** | **0.273** | **-** |
|  | MICE Control | **-** | 0.003 | 0.026 | **0.399** | **0.128** | 0.020 | **0.069** | **0.096** | 0.013 | 0.004 | 0.008 | **-** |
| Pleasure | HIIE-HV HIV | **-** | **-** | **-** | **-** | **-** | **-** | **-** | **-** | **-** | **-** | **-** | **0.078** |
|  | HIIE-LV HIV | **-** | **-** | **-** | **-** | **-** | **-** | **-** | **-** | **-** | **-** | **-** | **0.641** |
|  | MICE HIV | **-** | **-** | **-** | **-** | **-** | **-** | **-** | **-** | **-** | **-** | **-** | **0.448** |
|  | HIIE-HV Control | **-** | **-** | **-** | **-** | **-** | **-** | **-** | **-** | **-** | **-** | **-** | **0.138** |
|  | HIIE-LV Control | **-** | **-** | **-** | **-** | **-** | **-** | **-** | **-** | **-** | **-** | **-** | 0.027 |
|  | MICE Control | **-** | **-** | **-** | **-** | **-** | **-** | **-** | **-** | **-** | **-** | **-** | **0.311** |
| FEI | HIIE-HV HIV | **-** | **-** | **-** | **-** | **-** | **-** | **-** | **-** | **-** | **-** | **-** | 0.017 |
|  | HIIE-LV HIV | **-** | **-** | **-** | **-** | **-** | **-** | **-** | **-** | **-** | **-** | **-** | **0.060** |
|  | MICE HIV | **-** | **-** | **-** | **-** | **-** | **-** | **-** | **-** | **-** | **-** | **-** | **0.071** |
|  | HIIE-HV Control | **-** | **-** | **-** | **-** | **-** | **-** | **-** | **-** | **-** | **-** | **-** | **0.077** |
|  | HIIE-LV Control | **-** | **-** | **-** | **-** | **-** | **-** | **-** | **-** | **-** | **-** | **-** | 0.028 |
|  | MICE Control | **-** | **-** | **-** | **-** | **-** | **-** | **-** | **-** | **-** | **-** | **-** | **0.073** |

Data are presented as ***p*** (Shapiro-Wilk *p*-value). Values in bold are considered parametric.
